# Sidney John Hermann Griffiths

**Published:** 1933

**Authors:** 


### SIDNEY JOHN HERMANN GRIFFITHS, M.B., Ch.B., L.R.C.P., F.R.C.S.

It is with the most profound regret that we have to record the death of Mr. S. J. H. Griffiths. In the course of a full and vigorous activity he developed a septicæmia which was rapidly fatal on 25th February, 1933, after a short and trying illness endured by him with his usual courage and tenacity.

John Griffiths's surgical career, cut short as it was so soon, achieved an amazing success. The son of Mr. and Mrs. E. S. Griffiths, of Fairlawn, Portishead, he was born in Bristol on 21st July, 1898, and received his early education at St. Mary Redcliff School and privately. At the close of the War he entered on a medical course at Bristol University, taking his M.B., Ch.B., Bristol, in 1925, and winning the Committees'

Silver Medal for 1924–25. Then followed a postgraduate course at London University and London Hospital and Guy's Hospital, when he took his Fellowship. After a period as Senior Resident Officer at the Bristol General Hospital he became in 1929 a very welcome member of the Honorary Staff, and at once gained the complete confidence of his colleagues. Following this he was appointed to the Honorary Staffs of the Children's Hospital, the Homœopathic Hospital and the Winford Orthopædic Hospital. His ability as a surgeon both in the realms of orthopædics and of the thyroid gland was becoming widely recognized, and he carried on too with great energy a busy surgical consulting practice in Bristol. He was attended with loving regard by his house surgeons and students, who without exception unhesitatingly sought and much valued his friendly advice in matters of their career.

His intimate friends saw in Griffiths a very charming personality, a man of high principles with the gift of grasping the essentials of a problem and giving willingly straightforward advice on personal matters about which his opinion might be asked. A versatile and gifted raconteur, he would relate his experiences in realistic and humorous vein to the great entertainment of his listeners. He will be remembered with the sincerest affection. His life was a full one. Busy as he was, he took care never to let the private side of his practice usurp the time which he felt he should give in the interests of his patients at the various hospitals to which he was attached. His living, indeed his life's work, was based on his care for the welfare of others, and the comfort of his patients was always uppermost in his mind.

His death during the course of his rapid ascent is truly a tragic one. It leaves his friends with a sense of great personal loss, his colleagues with a gap most difficult to fill. Bristol and the West of England must lament a surgeon whose ambition was to confer upon the community widespread benefit and relief in the specialities which he practised.

